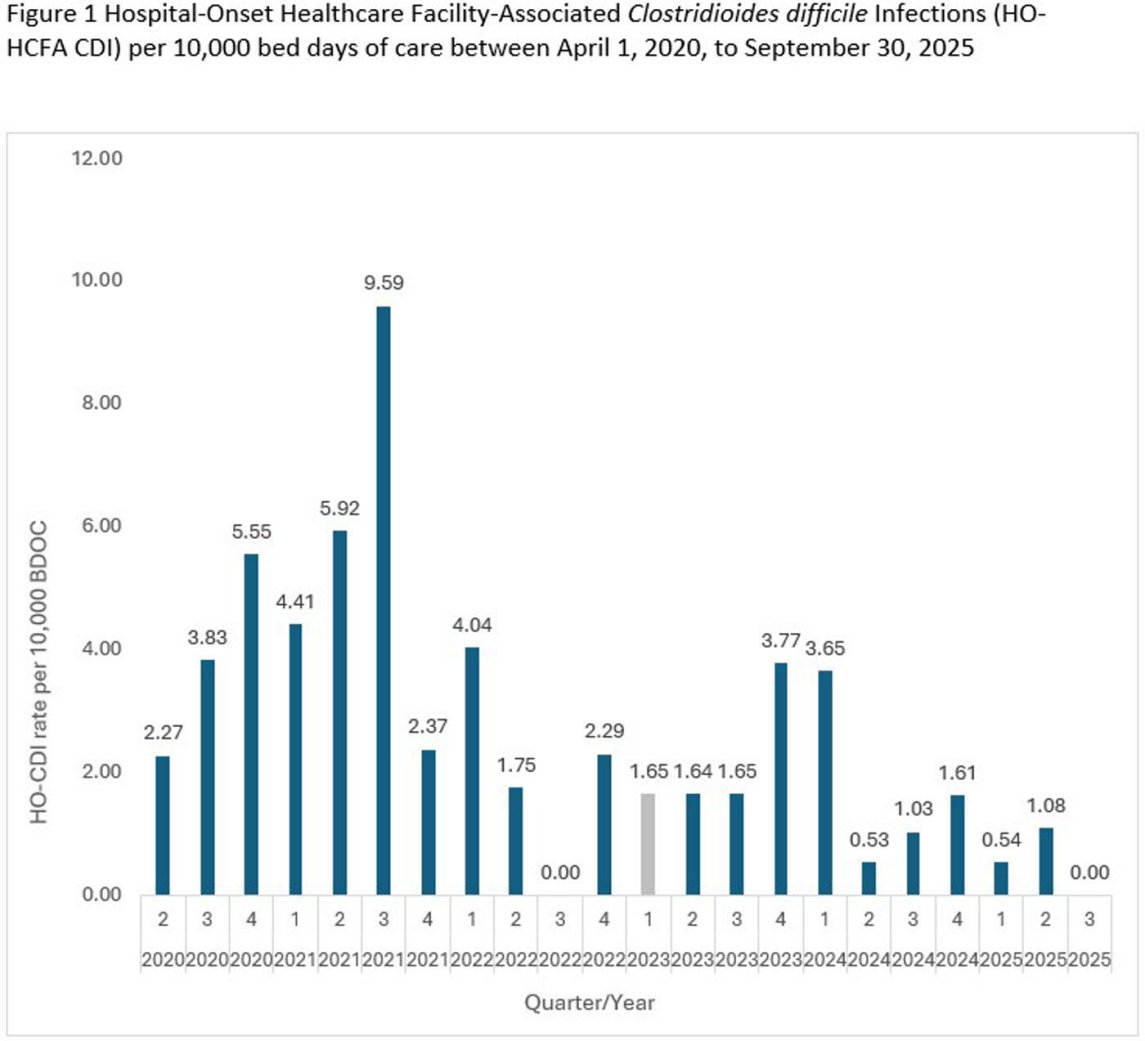# 135 PICC and Midlines: Evaluating Lumen Selection Appropriateness and Associated Complications

**DOI:** 10.1017/ash.2026.10545

**Published:** 2026-06-23

**Authors:** Reshma Narain, Linda Yang, Marcus Kouma, Jessica Guastadisegni, Islamia Omoola, Sherry R. Reid, Madhuri Sopirala, Parkland Health, Reuben Arasaratnam, Donald Storey, Ikwo Oboho

**Affiliations:** 1 University of Texas Southwestern Medical Center; 2 VANTHCS; 3 VA North Texas; 4 Department of Veteran Affairs; 5 Veterans Affairs North Texas Health Care System; 6 UT Southwestern; 7 Dallas VA Medical Center; 8 VA North Texas Health Care System/UT Southwestern University Medical Center

## Abstract

**Background:** Clostridioides difficile Infection (CDI) is one of the leading causes of hospital-acquired infections. The Veterans Health Administration launched a national CDI prevention initiative in 2012 that bundled core practices such as environmental cleaning, strict hand hygiene, and contact precautions. In contrast to prior studies which have noted a decline in CDI rates during the COVID-19 pandemic, rates of hospital-onset (HO)-CDI increased at our Veterans Administration Hospital, and we sought to address this issue with a multi-pronged approach. **Methods:** We assessed multi-modal approaches implemented to reduce HO-CDI rates at Veterans Affairs North Texas Health Care System (VANTHCS), a large, academic VA medical center from 2021 to 2025. This approach included laboratory changes to CDI testing, updates to infection prevention practices, and antimicrobial stewardship program (ASP) rounds for prospective audit and feedback for antibiotics (including those considered high-risk for CDI by the National Healthcare Safety Network). Our primary objective was to assess the impact of these interventions on the incidence of HO-CDI, comparing HO-CDI rates before (Q2 2020–Q4 2022) and after (Q2 2023–Q3 2025) their implementation. HO-CDI was defined as a LabID event collected from an inpatient location < 3 days after admission (i.e., on or after hospital day 4), and rates were reported as number of HO-CDI/10,000 bed days of care (BDOC). Cases were extracted from the Computerized Patient Record System. **Results:** In October 2021, VANTHCS switched from a standalone nucleic acid amplification test (NAAT) to a 2-step algorithm wherein an initial NAAT was reflexed to a toxin immunoassay if positive. The Infection Prevention and Control Program developed standard operating protocols for room cleaning and disinfection, and hand hygiene was reinforced. In February 2023, the facility ASP implemented thrice-weekly rounds. Institutional antimicrobial guidelines were developed for common infections, and educational presentations were delivered to providers, using illustrative cases to highlight key stewardship principles. ASP team members audited 2,282 patient charts from April 1, 2023, through September 30, 2025. Interventions were made for 1,215 (53%) patients, and prescribers accepted 972 (80%) recommendations. Common interventions consisted of either antibiotic discontinuation or de-escalation in 74% of patients. Mean quarterly high-risk CDI antibiotic use declined from 95 to 71 per 1,000 BDOC (25%, p<0.01), and HO-CDI rates declined 59% from 3.81 (pre) to 1.55 (post) infections per 10,000 BDOC. **Conclusion:** A multipronged approach of reinforcing IPC practices, 2-step testing, and targeted feedback for antibiotics use led to significant decreases in HO-CDI rates.

**Figure:**